# How a Medical Association Can Make a Difference in a Crisis Situation

**DOI:** 10.1097/HS9.0000000000000765

**Published:** 2022-08-09

**Authors:** Nathalie van Havre, Enrica Orsini, Antonio Almeida, John Gribben, Natacha Bolanos, Robin Doeswijk, Gianluca Gaidano, Samir Mouhssine, Konstanze Döhner, Kirsten Grønbæk, Kimmo Porkka, Elizabeth Macintyre

**Affiliations:** 1 European Hematology Association Executive Office, The Hague, The Netherlands; 2 Hospital da Luz, Lisboa, Portugal; 3 Centre for Haemato-Oncology, Barts Cancer Institute, London, United Kingdom; 4 Lymphoma Coalition Europe, Madrid, Spain; 5 Division of Hematology, Department of Translational Medicine, Università del Piemonte Orientale, Novara, Italy; 6 Department of Internal Medicine III, Ulm University Hospital, Ulm, Germany; 7 Department of Haematology, Rigshospitalet, Copenhagen, Denmark; 8 Department of Hematology, Helsinki University Hospital Cancer Center, Helsinki, Finland; 9 Hematology, Université Paris Cité and Necker Hospital, Assistance Publique—Hôpitaux de Paris, France

## EHA JOINS FORCES FOR UKRAINE

On May 4, 2022, Maria (not her real name), a 44-year-old Ukrainian woman affected by acute lymphoblastic leukemia, reached the Maggiore Charity Hospital (Ospedale Maggiore della Carità) in Novara, Italy. Maria had been suffering from acute lymphoblastic leukemia since 2019, and by February 2022, she was receiving maintenance therapy after her first complete remission when the war broke out, putting an abrupt stop to her follow-up care. After being moved to Italy, she is now continuing her treatment with human leukocyte antigen (HLA)-typing and donor search, in case a hematopoietic stem cell (HSC) transplantation is necessary.

Maria is one of the patients enrolled in the European Hematology Association (EHA)-coordinated program launched in April 2022 to support Ukrainian hematologists and their patients. This program provides economic support for humanitarian organizations, drugs, and medical supplies, as well as relocation for patients suffering from hematological diseases (Box 1). In this report, we will describe the design and implementation of the program, to encourage others to act, and to demonstrate what medical associations can do in a crisis situation.

## MARIA’s STORY

Maria is from Dnipro, in eastern Ukraine, where she worked as a dentist in the Dnipro Medical Academy. After the start of the war, her hematologist included her in a list for treatment abroad. Thanks to the lymphoma coalition (LC) and the Fondazione Italiana Linfomi (FIL), she has reached the Novara Hospital. Leaving behind her husband, father, and two brothers, one of whom is currently a soldier in the Ukrainian Army, she arrived in Novara with her mother and her 11-year-old daughter, who is attending school online. Besides thanking the LC and the local hematology team for welcoming her, she is particularly grateful to *AIL Novara* (a local charity devoted to leukemia care and research) “for immediately donating a laptop to my daughter, thus allowing her to attend school at a distance in Ukraine to complete her school year. And I have also been very happy to receive an Italian SIM card, which makes me able to call my family in Ukraine. Videocalls provide an essential way of feeling at home with friends and family, despite thousands of kilometers of separation.”

From the clinical point of view, her Italian doctors also had some issues to sort out. As one of Novara’s physicians observed: “The lack of molecular diagnosis (only the immunophenotype was provided), the language barrier (the patient can speak only in Ukrainian and Russian, so we had to consult a cultural mediator and a translator app), and the fact that she was not vaccinated against SARS-CoV-2 (we administered to her the first vaccine dose) posed several practical problems that, however, have been finally solved.”

It is important to highlight that Maria’s relocation was a collaborative effort. Apart from organizing the move and the clinical care, support has also been arranged for her family members. They are currently fully hosted by AIL in one apartment, and once Maria is discharged from the hospital, she will be reunited with them. The AIL volunteers, in cooperation with the hematology team, are ensuring that Maria’s and all other patients’ loved ones are provided with all their daily needs, visiting them regularly, and organizing social activities to overcome the feelings of war and illness in a foreign country.

## EHA’s ACTIONS FOR UKRAINE

When Russia’s invasion of Ukraine started on February 24, 2022, the world was shocked by the reality of a brutal war inside Europe. The consequences of this reality became clearer in the following weeks, leading every person and organization in Europe to ask themselves how they could help alleviate the suffering of the Ukrainian people.

As a medical organization, EHA felt compelled to provide active support to the Ukrainian hematological community, including clinicians and their patients, who were directly or indirectly impacted by the war.

On March 1, EHA published a statement of support for Ukraine on its website, reinforced on March 23 in collaboration with the French Society for Hematology and endorsed by 22 European hematologic National Societies (NSs). Although an important, coordinated response to the war by European medical associations, the statement was only a first step, and thus an entire program was put in place to assist Ukrainian hematologists and patients.

The actions planned (Box 1) focused on three forms of humanitarian assistance: (1) Provision of economic support for organizations and non-government organizations (NGOs) actively involved in medical aid, (2) Coordination of efforts to alleviate the medication shortage in Ukraine, and (3) Organization of transport into other European countries for hematology patients not able to receive necessary treatments within Ukraine.

1. Financial help is, of course, the first line of support organizations may provide: the EHA Board chose to actively contribute to the activities of Médecins sans Frontières (MSF). MSF has been present in Ukraine since the start of the conflict, providing essential emergency aid, medical supplies, and equipment. The Ukrainian health system is modern and well organized, but no health system can absorb the huge increase in demand for care following the months of destruction that Ukraine is experiencing. By May 5, MSF had more than 570 people working in Ukraine and had distributed more than 460 tons of medical supplies to hospitals. Another substantial amount went to Helping to Leave, a small NGO composed entirely of volunteers, whose medical department, in collaboration with LC, worked to transport patients out of Ukraine, particularly hematology and oncology patients. They not only transport people, but provide food, clothes, and psychological counseling along the way. Helping to Leave brought more than 10,000 people out of Ukraine throughout the evacuation process. Financial help was also provided to the LC to cover the needs of the hematological patients’ relocation program (see later).
2. The acute shortage of medicine and medical equipment, caused by distribution difficulties and the widespread, destructive bombings, is one of the main problems facing the Ukrainian medical system. On top of the needs of those directly involved and injured in the conflict, this situation can be life-threatening for people needing care for medical conditions, including hematological conditions. EHA has concentrated its efforts on coordinating the delivery of supplies to Ukrainian hospitals (MSF, LC), and institutions (EU, World Health Organization [WHO] Emergency Committee), via the European Cancer Organisation-American Society of Clinical Oncology (ECO-ASCO) Special Network for Ukraine. The aim was to avoid sending similar lists of supplies to the same institution as different groups, and at the same time creating connections with pharmaceutical industries and other potential sponsors interested in providing materials.
3. The war has had a devastating impact on hospitals due to supply shortages and deteriorating care conditions. This has raised the need to protect patients suffering from hematological conditions, especially those requiring urgent intervention and chronic treatment. EHA worked intensively on organizing a safe escort out of Ukraine for these patients and their families, a complex process requiring close contacts with Ukrainian hematologists, identification of European institutions available to receive them, and a digitized inventory of medical needs and resources available at the participating centers. To this end, EHA worked closely with the national hematology societies, to identify single entry points and connect these (emerging) national hubs with hematologists in Ukraine and Poland and with LC, to support them in their search for treatment options for Ukrainian patients, and built a website with an extensive dataset including patients’ clinical characteristics and medical records (diagnosis, treatments/protocols, special care needs), and the details of services offered in each hospital. Maria’s story is an example of how this system worked. The website is managed by LC, through a Ukrainian refugee specifically appointed for this position and has been developed as an adaptable tool to be used later in other emergencies, as necessary. EHA provided financial help to LC for the hiring of this position and other figures necessary for the project’s administrative support.

The Ukrainian Hematology Association established a list of adult patients needing transport to hematology units in Europe. By the end of May 2022, around 80 of them (out of 250) had been transported from Ukraine to hospitals within Europe. Each patient is assigned a level of urgency (“high priority,” “medium,” and “low”), and transfers are ongoing.

Finally, we must remember that a substantial effort will be needed at the end of this war to rebuild Ukraine’s scientific and medical infrastructure. In the meantime, the scientific community should support Ukrainian physicians and scientists, guaranteeing the continuous education of young generations and offering fellowships and educational opportunities wherever possible. To this aim, EHA offered free EHA membership for 2022 to all Ukrainian hematologists and researchers, including free access to the many education courses present in the EHA Campus, and free access to the EHA2022 Hybrid Congress and all other upcoming 2022 meetings.

## WHAT MAY BE LEARNED?

The entire Ukraine program was designed with the intent to provide support for hematologists and their patients in a crisis situation, fostering collaboration between different national and international institutions. This report highlights the intense work and commitment of involved associations, as well as the impact this commitment may have on the lives of people in need. It also made clear that substantial steps must be undertaken to ensure the quick and effective implementation of the planned actions in a rapidly evolving and highly dangerous situation.

One of these critical steps was to ensure continuous communication within and between all organizations involved in each task. Emergency situations spur the justified urge to do something, and to do it fast. However, although more immediate needs can be obvious and prompt immediate action, attention must be given also to less visible needs of the involved population. From the medical point of view, aside from emergency care for people directly involved in the fighting, a war also calls for action in favor of chronically ill people, and of people living in non-fighting zones but who are exposed to shortages and collateral damages. Effective coordination reduces the risk of duplicating already-received aid and provides a more comprehensive overview of potential actions to ensure aid offered actually reaches those in need. The coordinated work of international associations like EHA, and of national and local ones, can be a critical factor in this process.

As such, appointing a small number of dedicated people inside each participating association also proved imperative. In global emergencies such as the present one, a considerable number of organizations can be involved, including large ones like the WHO and European agencies. A risk that became evident during the planning and implementation of the Ukraine support program was to have too many actors at play, leading to long, inconclusive meetings, and drawn-out committee appointments. Each participating organization should define the scope and limits of its involvement, and delegate the actual design and implementation of their actions to one or a small group of people.

While relocating hematology patients to hospitals outside Ukraine, some crucial factors became evident, exemplified in Maria’s story: the availability of European national health systems to cover medical expenses and to provide refugees with a comprehensive health coverage; establishing systems able to overcome language barriers (including professional interpreting and cultural mediators, or else digital translation tools); and, providing social support and arranging the stay of patients’ relatives. In this context, the transmission of patients’ health information and medical records from their Ukrainian caring physicians to the host structures, passing through the digitization of medical data in a single web database, was of major importance to build health pathways and ensure a fast and optimal match of needs and local facilities. A map of the medical structures and other services mobilized for the care of refugees, including services offered and special care possibilities, was built and made available to all involved organizations, together with an operational directory of medical conditions and special needs to classify potential relocation candidates. The work, including the need for a General Data Protection Regulation (GDPR)-compliant system, was done with the help of a dedicated Ukrainian refugee, and remains a valuable tool for potential future crises.

Finally, essential in all this was the role of patients’ organizations (often the first to mobilize and to advocate for hematological patients) and of volunteers’ associations (such as AIL and FIL in Italy), willing to provide extra support to patients and relatives which national health systems were unable to offer.

## CONCLUSIONS

The situation in Ukraine is still rapidly evolving, with dramatic escalation scenarios not yet outside the range of possible outcomes. What is clear at this point is that the conflict is likely to be prolonged, with more victims and further suffering for the entire Ukrainian population in the future. Each organization can and should consider how it can help, within its capabilities and focus of activity.

As the leading hematology organization in Europe, EHA is striving to support the Ukrainian hematological community and their patients. A great deal of work, summarized in this paper, has already been done, and more is planned. The action undertaken to this point will provide valuable lessons for future ones. We hope that sharing these efforts and these lessons, as we attempted to do here, will encourage other organizations to act and add their contribution to mitigate the devastating effects of this crisis on the Ukrainian people.

**Box 1. Overview of the EHA’s actions planning for Ukraine**

-   March 1, 2022: **Statement of support** to Ukraine
-  **Free EHA membership** and free access to EHA congress and campus for all Ukrainian hematologists

**1**. Economical support

- **Donation to Medecins sans Frontières**, aimed at:
  º Field hospitals in Ukraine and neighboring countries
  º Supplying medication within Ukraine (including for hematology)
  º Support for refugees
- **Donation to Helping to Leave** (helpingtoleave.org), an NGO committed to:
  º Providing consultation to people who need to evacuate Ukraine
  º Guiding people through the evacuation process
  º Providing financial and psychological assistance and transit to a safe area
  º Focus on oncohematological patients in collaboration with LC

**2**. Actions on medication shortage
  º Coordinated interaction with institutions (WHO, EU) via the ECO-ASCO Special Network for Ukraine, and alignment with European Society for Medical Oncology (ESMO) and European Society for Blood and Marrow Transplantation (EBMT):

▪ Cooperation through this network has helped prevent multiple parallel, duplicate, or even competing actions on medicine shortages. Through this network, the shortage list initially prepared by the LC in collaboration with Ukrainian hematologists has found its way to the WHO Emergency Committee. The list has also been shared with EHA’s sponsors interested in providing aid
  º Collaboration with MSF and LC:
▪ Shortage list of hospitals and drugs needed shared with MSF
▪ MSF active in delivering needed supplies to hospitals
  º Contacts made with pharma (including list of drugs needed) to encourage them to collaborate with EHA and ECO-ASCO
**Coordination care** with LC for all hematological patients:
  º Contact with hematological National Societies (NSs) to establish single entry points or contact person
  º Development of a website (managed by LC) dedicated to an inventory of both hospitals that can support refugees, and patients in need of care:
  ▪ Built by volunteers
  ▪ Extensive dataset that includes protocols/treatments and needs on patient side and details of services/special care offered in each hospital
  ▪ Adaptable for use in other emergencies
  º Financial help to LC:
▪ For administrative support. EHA identified a Ukrainian refugee to take this position. This is a position funded by EHA but the person will be employed by LC
▪ For direct support to LC office. Again, a Ukrainian refugee in Germany previously working at the Ukrainian Cancer organization has taken this position
  º Zivver account (secure file transfer GDPR and patient confidentiality compliant) established for LC

## ACKNOWLEDGMENTS

A sincere thank you to Aidan Haigh for his diligent proofreading and editing of this article.

## AUTHOR CONTRIBUTIONS

NvH and EO contributed to the conception and design of the manuscript and wrote the first draft of the manuscript. All authors read, revised, and approved the final manuscript.

## DISCLOSURES

The authors have no conflicts of interest to disclose.

